# Evaluation and Management of Patients with Noncardiac Chest Pain

**DOI:** 10.1155/2008/708218

**Published:** 2009-04-22

**Authors:** C. Shekhar, P. J. Whorwell

**Affiliations:** Neurogastroenterology Unit, Wythenshawe Hospital, Manchester M23 9LT, UK

## Abstract

Up to a third of patients undergoing coronary angiography for angina-like chest pain are found to have normal coronary arteries and a substantial proportion of these individuals continue to consult and even attend emergency departments. Initially, these patients are usually seen by cardiologists but with accumulating evidence that the pain might have a gastrointestinal origin, it may be more appropriate for them to be cared for by the gastroenterologist once a cardiological cause has been excluded. This review covers the assessment and management of this challenging condition, which includes a combination of education, reassurance, and pharmacotherapy. For the more refractory cases, behavioral treatments, such as cognitive behavioral therapy or hypnotherapy, may have to be considered.

## 1. INTRODUCTION

Noncardiac chest pain (NCCP) is defined as the presence of pain resembling angina in the absence of evidence of any coronary artery disease [1, 2]. The pain, which may be relieved by glyceryl trinitrate (GTN), can radiate to the arms or neck although it can be very difficult to separate NCCP from true cardiac pain [3]. However, a distinguishing feature which can be useful is the radiation of the pain to the back which is not so common in angina [4]. It is important that cardiac disease is excluded at least by an exercise stress test and, in many cases, it may be necessary to undertake an angiogram. The latter is especially useful in refractory cases so that physicians can be secure about their reassurance as well as being confident in their therapeutic approach. Nothing undermines management more than endlessly repeating the cardiogram “just in case.”

## 2. ASSESSMENT

Because of its close relationship to the heart, the esophagus is often incriminated in NCCP but this may not always be the case. Gastroesophageal reflux disease (GERD) is probably the commonest cause of pain arising from the esophagus and this can be assessed by pH monitoring although there has been a recent trend to replace this with a therapeutic trial of a proton pump inhibitor (PPI) [5]. Gastroscopy is also worthwhile [6] and perhaps we should be considering esophageal biopsy more frequently in the light of the increasing number of reports of eosinophilic esophagitis [7], as we know little about the contribution of this problem to chest pain. Routine blood tests are unlikely to be helpful although barium studies of the esophagus may raise the suspicion of a motility problem. It is possible to record the motility of the esophagus or its sensitivity to either mechanical or chemical (e.g., acidic) stimuli [8, 9] and although useful for research purposes, this approach has not found a great deal of utility in the routine investigation of NCCP. With respect to motility recording, a number of different contraction abnormalities of the esophagus have been described, such as the nutcracker esophagus, although the temporal relationship of these events to symptoms has not always been clear cut. More recently, there has been increasing interest in the phenomenon of nonacid esophageal reflux [10] but its role in NCCP has not yet been defined. This problem can be identified by esophageal impedance monitoring [11] although at present this technique tends to be confined to research centers.

Another important concept, especially from a therapeutic point of view, that is common to all functional gastrointestinal disorders, is the notion that the central processing of noxious stimuli from the gut is disturbed [12]. Thus, how the patient perceives and reacts to their pain is critical and there is some evidence that this may be a problem in patients with NCCP [13–16]. These patients also exhibit a higher prevalence of psychopathology than those with angina and a simple assessment by an instrument such as the hospital anxiety depression scale (HAD) [17] can be very useful. A dietary evaluation is also worthwhile as certain foods or drinks may exacerbate the pain and it has been shown that alcohol and nicotine can affect lower esophageal sphincter function [18, 19]. Furthermore, it should be remembered that a range of medications might exacerbate gastrointestinal symptoms in general as well as esophageal symptoms in particular, so a drug history is helpful.

## 3. MANAGEMENT

Once a cardiological cause for the pain has been ruled out, an assessment along the lines already described should be undertaken. Reassurance is crucial but these patients are notoriously resistant to this because of the lethal connotations of chest pain and the fear that something might have been overlooked.

A trial of a PPI should be the first pharmacological step and these may be required at high doses such as omeprazole 40 mg twice a day or equivalent. If the patient responds, then GERD-related chest pain is the most likely diagnosis and maintenance treatment may be required. Failure to respond to a PPI suggests that acid reflux is not the problem although obviously this does not exclude nonacid reflux. In the absence of reflux, it is usually assumed that the problem may be due to a disorder of motility or sensation. On the assumption that esophageal contractions may be contributing to the pain, a variety of treatments aiming at relieving spasm have been evaluated. Drugs such as GTN, long acting nitrates, and a variety of calcium channel blockers have been tried with variable results and some problems with side effects [20, 21]. Nifedipine has probably received the most attention, probably because of the demonstration of an effect on esophageal motility [22, 23] and this drug is sometimes worth a trial although there is no convincing evidence of efficacy. 

There is a general consensus that antidepressants, particularly of the tricyclic variety, are valuable in irritable bowel syndrome although the data supporting this view are rather old [24, 25]. Not surprisingly, therefore, a variety of tricyclic agents have been tried in NCCP although, as with the IBS studies, these studies are somewhat dated. However, the results have been consistently positive suggesting that these drugs have a definite role in managing the condition [26–28]. It is a usual practice in functional gastrointestinal disorders to use relatively low doses of tricyclic antidepressants starting with amitriptyline 10 mg daily or equivalent. There is evidence that selective serotonin reuptake inhibition can reduce chemical and mechanical sensitivity of the esophagus [29] although there has only been one study assessing the effect of this class of drug in NCCP, but this was encouraging [30]. Benzodiazepines have also been shown to possibly be beneficial [31, 32]. This is probably because of their anxiolytic properties as patients with NCCP have been shown to be prone to panic disorder [33–36]. Unfortunately, this group of drugs is out of favor at present because of their addictive potential. However, if patients can be trusted to take them on an “as necessary basis” when the pain is especially severe, we find that they have a role in NCCP and other functional gastrointestinal disorders, as long as they are used with caution. There has been recent interest in the possibility that theophylline might have a role in the treatment of NCCP [37], especially as it appears to increase the threshold to balloon distension-induced pain in the esophagus. In the one trial so far reported, the drug appeared to significantly relieve symptoms in a relatively small group of patients [38]. There have also been some reports that the injection of Botox might help spastic disorders of the esophagus [39] although there have been no formal controlled trials. 

Behavioral treatments are being increasingly used in functional gastrointestinal disorders and NCCP is no exception to this rule. There have been five studies of cognitive behavioral therapy (CBT) in this condition all of which have been positive [40–44]. Whether this form of treatment has a long lasting effect is not so certain although one of these trials suggested benefit may be sustained [38]. Another form of treatment that is gaining some acceptability in FGD's is hypnotherapy [45, 46] and we have recently reported a trial of this modality in a small group of highly selected patients with NCCP [47]. These results were very encouraging and we have subsequently shown that the beneficial effects appear to be long lasting [44, 48]. 

Thus, in summary, it is mandatory to exclude a cardiac cause for chest pain before undertaking appropriate gastrointestinal investigation and making a confident diagnosis of NCCP. Once this has been established, a trial of a PPI should be the next step. Subsequent investigation should be kept to a minimum and a variety of other medications can be tried although the tricyclic antidepressants are probably the most effective. If pharmacological approaches fail, behavioral treatments can be of value depending on their availability.
